# Late-window reperfusion in imaging-selected ischemic stroke: interpreting thrombolysis and mechanical thrombectomy trials

**DOI:** 10.3389/fneur.2026.1794076

**Published:** 2026-07-14

**Authors:** Levi Dygert, Yidan Shi

**Affiliations:** 1 Department of Neurology, Division of Stroke and Cerebrovascular Disease, NYU Langone Health, New York, NY, United States; 2 Department of Population Health, Division of Biostatistics, NYU Langone Health, New York, NY, United States

**Keywords:** acute stroke, ischemic stroke, ischemic/reperfusion injury, stroke, thrombectomy, thrombolytic therapy

## Abstract

Mechanical thrombectomy (MT) is superior to medical therapy for anterior circulation proximal large-vessel occlusion (LVO) stroke, including in imaging-selected patients treated 6 to 24 h after their last known well (LKW) time. Beyond 4.5 h, the role of intravenous thrombolysis (IVT) has become more relevant because many health systems lack the ability to provide timely thrombectomy access for eligible late-window patients. Recent late-window IVT trials, including TRACE-III and HOPE, have demonstrated benefit in imaging-selected patients in whom thrombectomy was not planned or not effectively deliverable, addressing a critical systems-of-care gap. We review these trials alongside emerging evidence and situate each within a systems-of-care framework, distinguishing bridging scenarios from settings where MT is unavailable or substantially delayed. However, we argue that their results should not be taken to imply therapeutic equivalence with MT, a comparison that is misleading on methodological grounds. No randomized, head-to-head late-window trial of IVT versus MT exists. Comparing treated-arm outcomes across these separate trials as a proxy for therapeutic equivalence is vulnerable to differences in baseline prognosis, occlusion site, imaging selection, access to rescue therapy, and control-group outcomes. In this Perspective, we argue that these trials should be read one at a time. Each treated arm should be judged against its own control group and against the clinical and angiographic profile of the patients enrolled, not against treated arms from trials that enrolled very different strokes.

## Introduction

Late-window thrombectomy trials have definitively established that selected patients with proximal anterior-circulation large-vessel occlusion (LVO) benefit substantially from mechanical thrombectomy (MT) compared with standard medical care. In the DAWN trial (6–24 h), functional independence, defined as a modified Rankin Scale (mRS) score of 0–2 at 90 days, occurred in 49% of patients treated with thrombectomy versus 13% with standard care, with similar rates of symptomatic intracranial hemorrhage (sICH) and mortality (1). In the DEFUSE 3 trial (6–16 h), functional independence occurred in 45% of patients treated with thrombectomy versus 17% with standard care, with lower mortality rates in the thrombectomy group and no significant difference in sICH (2).

The role of intravenous thrombolysis (IVT) beyond 4.5 h has become relevant again because access to thrombectomy remains uneven across and within countries, for reasons that include transfer dependence, rural geography, and resource constraints. Two recent randomized trials, TRACE-III with tenecteplase (3) and HOPE with alteplase (4), provided core evidence of late-window IVT in imaging-selected patients in whom thrombectomy was not accessible or not planned at enrollment.

The resulting debate is not only on whether late-window IVT is beneficial when MT is unavailable or delayed, but also about how its outcomes should be interpreted relative to late-window MT. Some commentary has emphasized that treated-arm functional outcomes in late-window IVT trials appear numerically similar to those in late-window MT trials. This comparison is misleading because treated-arm outcomes reflect both treatment efficacy and baseline prognosis, and these trials enrolled substantially different stroke populations.

In this Perspective, we review late-window reperfusion trials within a systems-of-care framework, discuss IVT in transfer-dependent systems and settings with pragmatic imaging constraints, and argue that treatment effects should be interpreted by anchoring outcomes to randomized control arms and enrolled population characteristics rather than by comparing treated-arm absolute outcomes across trials.

## Trial populations differ

### Late window reperfusion trials enrolled different populations

In the TRACE-III trial, patients with anterior-circulation large- or medium-vessel occlusion involving the ICA, M1, or M2 were randomized to receive tenecteplase 4.5–24 h after their last known well (LKW) time. Patients were perfusion-selected by CT perfusion and enrolled a median of ~12 h after their LKW. Baseline stroke severity was moderate (median NIHSS of ~10; group medians of 11 and 10, respectively) (3). Although TRACE-III applied the same late-window imaging selection principle as the DAWN and DEFUSE 3 trials, its population differed clinically and anatomically, with lower baseline NIHSS scores and inclusion of M2 occlusions, and the treated-arm outcomes should be interpreted accordingly.

The HOPE trial randomized patients to receive alteplase 4.5–24 h after LKW in perfusion-selected patients without an initial thrombectomy plan and did not require LVO as an inclusion criterion (4). Baseline NIHSS scores were ~10 (median 10; IQR 6–14 in the alteplase group and 6–15 in the control group). Proximal anterior LVO, defined conventionally as intracranial ICA and/or M1, accounted for only ~45% of each arm (84/186 and 85/186), substantially lower than in the DAWN and DEFUSE 3 trials. This directly limited cross-trial inference against thrombectomy trials that require proximal LVO by design.

### Late-window thrombectomy trials

The DAWN and DEFUSE 3 trials required proximal anterior circulation occlusion (intracranial ICA and/or proximal MCA) and enrolled patients with substantially more severe strokes, with median baseline NIHSS scores of 17 and 16, respectively, compared with ~10–11 in TRACE-III and ~10 in HOPE (1, 2).

Across these trials, “LVO” was not uniformly defined, the occlusion sites differed, and the baseline severity varied substantially. Because subgroup definitions and reporting also differ, aggregate trial-level comparisons cannot fully harmonize these populations. This heterogeneity reinforces the need to interpret late-window IVT evidence within each randomized trial rather than through informal treated-arm comparisons with MT trials (Table 1).

**Table 1:** Key differences in late-window reperfusion trial populations and outcomes.

Trial	Population	Median NIHSS	Occlusion requirement	Reperfusion strategy	Primary reported 90-day functional outcome
DAWN	Late-window proximal anterior LVO	17	ICA/M1	MT vs. standard care	mRS 0–2: 49% vs. 13%
DEFUSE 3	Late-window proximal anterior LVO	16	ICA/M1	MT vs. medical therapy	mRS 0–2: 45% vs. 17%
TRACE-III	Late-window LVO/MeVO without thrombectomy	~10	ICA/M1/M2	TNK vs. standard care	mRS 0–1: 33.0% vs. 24.2%
HOPE	Late-window perfusion-selected stroke, no initial MT plan	~10	LVO not required	Alteplase vs. standard care	mRS 0–1: 40.3% vs. 26.3%
TIMELESS	Late-window ICA/M1/M2, primarily MT-treated	~12	ICA/M1/M2	TNK vs. placebo, mostly before MT	mRS 0–2: 46.0% vs. 42.4%

Primary endpoints differ across trials: DAWN, DEFUSE 3, and TIMELESS report mRS 0–2 (functional independence), whereas TRACE-III and HOPE report mRS 0–1 (excellent outcome). Treated-arm percentages are therefore not directly comparable across rows.

## Control arm outcomes reflect different untreated natural histories

In randomized trials, treatment efficacy is estimated by comparing outcomes between the treatment and control groups within the same trial. Treated-arm outcomes alone can be misleading because they combine two distinct factors: the effect of the intervention and the baseline prognosis of the enrolled population. Baseline prognosis reflects the likelihood of recovery based on clinical severity, occlusion site, collateral status, and infarct core volume, independent of the assigned treatment. Therefore, a 45- to 50% rate of functional independence after treatment can have different meanings across trials. In a population with severe proximal LVO and poor untreated outcomes, it may reflect a large treatment effect. In a population with lower NIHSS scores, more distal occlusions, or no visible proximal occlusion, the same treated-arm outcome may reflect a smaller incremental benefit superimposed on a more favorable natural history.

While all these trials successfully selected slow progressors through imaging, the untreated natural history of this phenotype depends heavily on the severity of the initial deficit and the location of the occlusion, not on imaging selection alone.

- DAWN and DEFUSE 3 strictly enrolled patients with severe clinical deficits (median NIHSS scores of 16–17) and proximal anterior-circulation LVOs. Even with a favorable imaging profile (small core, large penumbra), leaving a proximal occlusion untreated in a patient with a severe baseline deficit is associated with a high likelihood of permanent disability. This is precisely what the observed control-arm rates reflect: functional independence was achieved by only 13–17% of untreated patients in the DAWN and DEFUSE 3 trials.
- TRACE-III and HOPE enrolled patients with moderate stroke severity (median NIHSS scores of ~10), and HOPE included a majority of patients without conventional proximal ICA/M1 occlusions. When selecting patients with less severe initial deficits and more distal or absent visible occlusions for favorable perfusion profiles, their smaller at-risk territories and more favorable collateral profiles may confer a more favorable untreated natural history. Consistent with this baseline profile, functional independence in the control arm was higher in the TRACE-III and HOPE trials than in the DAWN and DEFUSE 3 trials.

When late-window IVT is evaluated within randomized trials, rather than through informal comparisons of treated arms across different studies, the evidence supports consistent benefit for patients not undergoing mechanical thrombectomy. In a recent meta-analysis of eight randomized trials, treatment effects showed no evidence of statistical heterogeneity for excellent or good functional outcomes (5). Treated-arm absolute outcomes, by contrast, cannot be interpreted as evidence of therapeutic equivalence. A 45–50% functional independence rate in the treated arm can reflect a large treatment effect over a devastating natural history, as in DAWN and DEFUSE 3, or a smaller incremental effect over a comparatively favorable natural history, as in imaging-selected late-window IVT trials.

## Systems-of-care context and selection bias

Care-delivery context further shaped trial populations in ways that limit cross-trial inference. In the HOPE trial, enrollment required that no baseline thrombectomy plan existed due to factors that included perceived endovascular risk, low expected benefit, or substantial transfer delays, despite participating hospitals having thrombectomy capability (4). This design limited generalizability to systems where late-window MT is rapidly available and routinely pursued for eligible proximal LVO patients.

Access to MT is also influenced by cost and reimbursement. The RESILIENT trial highlighted that thrombectomy adoption in a middle-income public health system requires local demonstration of feasibility, efficacy, and cost-effectiveness (6). These factors help explain why some patients do not receive MT even when guideline-eligible and why “no planned thrombectomy” trial populations may reflect different care pathways than those in the DAWN and DEFUSE 3 trials.

## Bridging thrombolysis and transfer-dependent care

A key distinction when interpreting late-window IVT trials is whether IVT is being tested as the only reperfusion strategy available or as an adjunctive bridging therapy before MT. The TRACE-III and HOPE trials primarily addressed the former scenario by enrolling patients in whom thrombectomy was not planned, not accessible, or not effectively deliverable. The TIMELESS and ETERNAL-LVO trials, by contrast, addressed MT-capable pathways in which IVT was added before anticipated thrombectomy. When rapid progression to MT is expected, the incremental opportunity for IVT to improve outcomes may be smaller. In the TIMELESS trial, 77.3% of patients underwent thrombectomy, and tenecteplase did not improve the primary ordinal mRS outcome despite similar rates of functional independence, symptomatic intracranial hemorrhage, and mortality (7).

However, transfer-dependent systems create a distinct biological window in which the thrombolytic agent has time to act while the patient is in transit. A multicenter cohort study of 584 patients transferred for MT from primary centers beyond 4.5 h found that IVT before transfer was associated with higher recanalization during transfer and better 3-month functional outcomes without safety concerns, supporting the plausibility of a late-window IVT benefit specifically in transfer workflows (8).

## Imaging availability and pragmatic selection

Advanced perfusion imaging is not universally available in emergency settings. The TWIST trial tested tenecteplase in wake-up strokes selected by non-contrast CT across 77 centers in 10 countries and did not demonstrate significant benefit on the primary ordinal mRS outcome (9). The TWIST trial enrolled patients with predominantly mild strokes (median NIHSS scores of 6), low MT rates, along with a minority of proximal LVOs, differing substantially from the perfusion-selected LVO populations in the TRACE-III and the late-window MT trials. These results reinforce the idea that CT-only selection strategies likely require dedicated trials in the specific healthcare contexts and stroke severities where they will be applied and that results from perfusion-selected trials should not be assumed to generalize to CT-only settings. The CHESTNUT trial is prospectively testing this approach in China under optimized non-contrast CT selection (10).

## Conclusions and systems-of-care implications

- First, treated arm outcome similarity should not be taken as evidence of late-window IVT-MT equivalence. Differences in baseline prognosis (e.g., NIHSS scores, proximal LVO prevalence, and vascular territory) and care pathways (e.g., MT availability, transfer delays, refusal, and non-pursuit) invalidate such comparisons. A head-to-head randomized trial is also ethically difficult to justify, given MT’s large proven benefits in eligible proximal anterior-circulation LVO patients.
- Second, when MT is available and indicated for proximal anterior LVO in the late window, it remains the preferred therapy given its substantial relative benefit over standard care in DAWN and DEFUSE 3.
- Third, late-window IVT has pragmatic value in several contexts: (i) perfusion-selected non-LVO stroke, where MT is not routinely pursued; (ii) patients with LVO or MeVO when MT is not locally available; (iii) patients in whom MT is expected to be substantially delayed because of transfer dependence; and (iv) patients in whom late-window IVT can be started before transfer, allowing thrombolytic effects to occur en route.
- Fourth, the net benefit of late-window IVT is best understood as smaller than MT in eligible proximal LVO phenotypes; however, it is still clinically meaningful in selected patients without effective MT access, with a tradeoff of higher sICH risk and no clear mortality harm in aggregated randomized data.

## What is next

Trial design is actively diversifying in late-window contexts, and each emerging trial addressed a distinct scenario within the framework outlined above:

- *Perfusion-selected late-window thrombolysis in MT-capable settings (LVO/MeVO)*: The CHABLIS-T II trial improved early reperfusion without sICH, but it did not improve 90-day clinical outcomes, consistent with the TIMELESS trial, which found that when thrombectomy is anticipated and delivered, the benefit of incremental IVT is limited. The 54.9% planned MT rate underscored that this trial addressed the bridging scenario rather than the no-MT-access scenario, for which the TRACE-III and HOPE trials applied (11).
- *Late-window thrombolysis in non-LVO stroke*: The OPTION trial showed that tenecteplase administered 4.5 to 24 h in perfusion-selected patients improved excellent functional outcomes compared with standard medical care (12). This extended the late-window IVT evidence base to non-LVO stroke patients, a population for whom the role of MT remains under active investigation. In this context, OPTION addressed a scenario in which IVT is the primary available reperfusion strategy.
- *Late-window thrombolysis in MT-capable pathways (bridging scenario)*: The ETERNAL-LVO trial was terminated early with no overall difference in the primary 90-day excellent outcome endpoint. However, a prespecified transfer subgroup signal was reported (13). The early termination limits interpretation, but the overall null result is directionally consistent with the findings of the TIMELESS trial: in settings where MT is delivered, the added benefit of IVT bridging is uncertain and may be concentrated in transfer-dependent workflows rather than in direct-to-thrombectomy care.
- *Posterior circulation and basilar occlusion*: the TRACE-5 trial demonstrated that tenecteplase administered within 24 h significantly improved functional outcomes in basilar artery occlusion, extending the late-window IVT evidence base to a posterior circulation population where MT access is similarly uneven, and the no-treatment prognosis is severe (14). By contrast, the ATTENTION-LATE trial found that tenecteplase bridging prior to thrombectomy in late-window basilar artery occlusion did not improve functional outcomes compared with thrombectomy alone (presented at ISC 2026; not yet peer-reviewed) (15), mirroring the anterior circulation pattern: IVT adds value when MT is unavailable, but not when thrombectomy is promptly delivered.
- *CT-only selection*: the TWIST trial tested a CT-only approach in wake-up stroke, but it enrolled patients with predominantly mild strokes with low MT rates and did not show significant benefit (9), highlighting that pragmatic selection strategies likely require dedicated trials in the specific healthcare contexts and stroke severities where they will be applied. The CHESTNUT trial is prospectively testing whether optimized non-contrast CT selection can address this gap in populations and settings where perfusion imaging is not routinely available (10).
- *Early-window bridging informing late-window assumptions*: The BRIDGE-TNK trial found that tenecteplase before thrombectomy in the early window improved 90-day functional independence (16). However, the benefit was modest, and the secondary outcomes were not consistently significant. Its lesson for late-window interpretation is that thrombolytic contribution depends strongly on workflow timing and that the biological window in which IVT can meaningfully act is shaped by how quickly MT follows.

The through-line across these trials is consistent with the interpretive framework we have outlined, namely that late-window IVT evidence is most clinically actionable in populations and systems where MT is not available, not indicated, or substantially delayed. Where MT is delivered promptly, the incremental benefit of IVT bridging remains uncertain and is not established by current randomized evidence.

## Data Availability

The original contributions presented in the study are included in the article/supplementary material, further inquiries can be directed to the corresponding author/s.
